# Effects of Hydrostatic Pressure and Cation Type on the Chloride Ion Transport Rate in Marine Concrete: An Experimental Study

**DOI:** 10.3390/ma17133195

**Published:** 2024-06-29

**Authors:** Huanqiang Liu, Xueqing Yang, Linhua Jiang, Keliang Li, Weizhun Jin

**Affiliations:** 1 School of Civil Engineering and Transportation, North China University of Water Resources and Electric Power, Zhengzhou 450045, China; liuhuanqiang@ncwu.edu.cn (H.L.); likeliang@ncwu.edu.cn (K.L.); 2 School of Information Engineering, North China University of Water Resources and Electric Power, Zhengzhou 450046, China; yangxueqing@ncwu.edu.cn; 3 College of Civil and Transportation Engineering, Hohai University, Nanjing 210024, China; hhulhjiang@163.com

**Keywords:** hydrostatic pressure, cation type, chloride ion apparent transport coefficient, chloride ion binding capacity

## Abstract

The effect of hydrostatic pressure and cation type on chloride ion transport in marine underwater concrete cannot be ignored. The study of the chloride ion transport behavior of concrete under the effect of hydrostatic pressure and cation type coupling can provide a basis for durability design and the protection of marine concrete. In this work, the chloride ion transport behavior of marine concrete in four common chloride salt solutions under different hydrostatic pressures is studied by a hydrostatic pressure test device developed by the authors. The results show that hydrostatic pressure and its action time significantly influence the chloride ion transport behavior in marine concrete; the higher the hydrostatic pressure of concrete, the faster the chloride ion transport rate. The longer the time, the more chloride ions accumulated in the same position, and the farther the chloride ion transport distance. Cation type has a certain influence on the transport process of chloride ions. Under the same test conditions, the chloride ion transport rate in a divalent cation solution is about 5% higher than that in a monovalent cation solution. The results also show that the chloride ion binding capacity under hydrostatic pressure is only 10~20% of that under natural diffusion. Using the test results, a predictive model of a chloride ion apparent transport coefficient based on the hydrostatic pressure and hydrostatic pressure action time corrected by a cation type influence coefficient is established.

## 1. Introduction

Marine concrete used in underwater construction not only bears structural loads but also the hydrostatic pressure that increases with water depth [1,2]. The chloride ion erosion of buildings in marine environments is the main factor leading to premature structural damage and early retirement from service [3,4,5,6], and its transport rate in concrete is known to depend on hydrostatic pressure. A series of studies have been carried out on the effects of hydrostatic pressure on structural damage and chloride ion transport in concrete. Li et al. [7,8,9] reported that increasing hydrostatic pressure promoted the damage and porosity of underwater concrete. Zhao et al. [10,11,12] confirmed that high hydrostatic pressure damaged marine concrete structures and influenced the chloride ion transport behavior. Alfatlawi et al. [13] believed that when hydrostatic pressure and cracks coexist, the effect of hydrostatic pressure on the chloride ion transport behavior was stronger than that of cracking. Wei et al. [14] studied the influence of the chloride ion transport behavior of UHPC under different hydrostatic pressures, and the results showed that with the increase in hydrostatic pressure, the chloride ion concentration and chloride ion apparent diffusion coefficients at the same depth increased, and the effect was more significant at 2 MPa. Park et al. [15,16,17,18] obtained a conclusion similar to Wei’s study. According to the study by Yoo et al. [19], when the hydrostatic pressure was lower than 0.15 MPa, the water flow in concrete was laminar. According to Chen et al. [9,20], the chloride ion diffusion behavior under hydrostatic pressure did not follow Fick’s law. Jin et al. [21,22,23] studied the transport behavior of water and chloride ions in concrete under hydrostatic pressure. The results showed that the transport depth of chloride ions was only 53% of that of water, and the chloride ion transport coefficient under hydrostatic pressure was 500–600% of that of natural diffusion. It is not difficult to see that the current research mainly focuses on the qualitative analysis of the effects of hydrostatic pressure on concrete structural damage and chloride ion transport behavior and rarely involves quantitative research on the chloride ion transport coefficient and binding capacity of concrete under hydrostatic pressure.

Alternatively, the effect of cation type on the behavior of chloride ion transport has been also studied by several scholars [24,25,26]. Kondo et al. [27] and Ushiyama et al. [28] were early scholars who studied the effect of cations on the chloride ion transport behavior, and their research believed that bivalent cations had a greater effect on chloride ion transport than monovalent cations. GjrØv et al. [29] showed that the chloride ion diffusion rate of the CaCl_2_ solution was much faster than that of the NaCl solution when the chloride ion concentration was the same. Feldman [30] noticed that the cation type had a great influence on the chloride ion diffusion and migration process and summarized the studies by Kondo and Ushiyama. Arya et al. [31] found that the chloride ion binding capacity in concrete was much better in the CaCl_2_ or MgCl_2_ diffusion source solution than in the NaCl solution. Yue et al. [32] also showed that Ca^2+^ has a stronger ability to bind chloride ions than Na^+^. Subsequent studies showed that cation types could be ranked by their effect on chloride ion binding capacity as Ca^2+^ > Mg^2+^ > Na^+^ ≈ K^+^ [33,34]. Chu et al. [35,36] found that Mg^2+^ and Ca^2+^ had a more significant effect on chloride ion binding ability than Na^+^ and K^+^ through differential thermogravimetric tests. Geng et al. [37] believed that the influence of bivalent cations on the surface charge of C-S-H was greater than that of monovalent cations, and the surface charge significantly changed the absorption capacity of C-S-H for chloride ions. Liu et al. [38] used the RCM method and the natural diffusion method to study the influence of cation types on chloride ion diffusion. The results showed that the chloride ion diffusion coefficient of fly ash concrete is mainly affected by the valence state of cations, and the higher the valence state was, the greater the diffusion coefficient was. It is not difficult to find that there are more results on the effect of cation types on chloride ion transport behavior by the natural diffusion method, but there are few results on the effect of cation types on the chloride ion transport behavior under hydrostatic pressure.

Seawater contains Na^+^, K^+^, Ca^2+^, and Mg^2+^ cations [39], so the transport process of chloride ions in marine concrete is not only affected by hydrostatic pressure but also different types of cations in seawater. Investigating the chloride ion transport behavior under the coupling of hydrostatic pressure and cation type provides the research basis for the durability design of marine concrete. This study developed a hydrostatic pressure test device to carry out the chloride ion transport test under the coupling of hydrostatic pressure and cation type. The experimental results obtained were quantitatively analyzed. A calculation model of the chloride ion apparent transport coefficient under hydrostatic pressure was established and was modified by the cation type influence coefficient.

## 2. Chloride Ion Transport Equation under Hydrostatic Pressure

In natural diffusion, Fick’s law is often used to describe the chloride ion transport behavior. When subjected to hydrostatic pressure, the transport flux includes diffusion and convection fluxes. The transport flux can be represented by Equation (1), and the analytical solution has been derived from Equation (2) in [40].

(1) $$J_{i}=-D_{i}\nabla C_{i}-C_{i}v$$

(2) $$C_{i,x}=C_{i,0}+\frac{\left(C_{i,s}-C_{i,0}\right)}{2}[erfc(\frac{x-vt}{2\sqrt{D_{P,i}\cdot t}})+e^{\frac{vx}{D_{P,i}}}erfc(\frac{x+vt}{2\sqrt{D_{P,i}\cdot t}}]$$

where J_i_ is the *i*-th ion flux, mol/(m^2^·s); *D*_i_ is *i*-th ion diffusion coefficient, m^2^/s; *C_i_* is i-th ion concentration, mol/L; v is the convection velocity, m/s; t is time, s; x is i-th ion transport distance from the intrusion surface, m; *C_i,s_* is the *i*-th ion surface concentration, mol/L; C_i,0_ is the *i*-th ion initial concentration, mol/L; and *C_i,x_* is the *i*-th ion concentration at position *x*, mol/L.

Alternatively, parameter *D_P,i_* in Equation (2), linking the diffusion and convection fluxes, was referred to as the *i*-th ion apparent transport coefficient [9,17]. Accordingly, this study also defined D_P,Cl_ as the “chloride ion apparent transport coefficient”, m^2^/s.

## 3. Materials and Tests

### 3.1. Materials

In this study, ordinary Portland cement P·O 42.5 was used to prepare concrete. Its chemical compositions are listed in Table 1.

The fine aggregate was machine-made sand, which has a fineness modulus and apparent density of 2.6 and 2.65 g/cm^3^, respectively. The coarse aggregate was graded broken stone with a particle size of 5~25 mm and an apparent density of 2.72 g/cm^3^. The water–cement ratio of the concrete was 0.5. The amount of raw material per cubic meter of concrete was 310 kg of cement, 750 kg of fine aggregate, and 1120 kg of coarse aggregate. The compressive strength of the concrete at 28 d was 33.5 MPa.

### 3.2. Tests

**Figure 1:**
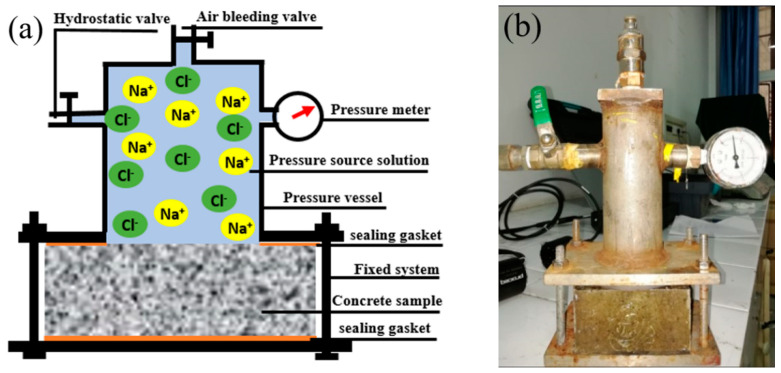
Schematic diagram (**a**) and the object of the hydrostatic pressure device (**b**).

The hydrostatic pressure of marine concrete acts vertically on the contact surface, so the motion of chloride ions under this condition can be regarded as one-dimensional transport. To simulate the hydrostatic pressure of marine concrete, a hydrostatic pressure device was designed and produced, and a schematic diagram and photo are shown in Figure 1. The test equipment was mainly made of a 304 stainless steel plate and seamless steel pipe welding. The diameter of the source solution storage chamber was 70 mm, the height was 200 mm, the width of the gasket was 15 mm, and the accuracy of the pressure meter was 0.01 MPa.

A hydrostatic pressure test was carried out using the above device, and the concrete blocks with sizes of 50 mm × 100 mm × 100 mm were used and obtained by cutting the concrete blocks used for compressive strength. After 2 weeks of curing, the two end faces, including the formed surface, were cut to ensure that the two end faces were smooth, easy to install and seal the device, and that the test results were not affected by the formed surface. All specimens were cured at (20 ± 2) °C and a relative humidity of over 95% for at least 28 d before they were used for the chloride diffusion experiments under hydraulic pressure. The compressive strength of the concrete at 28 d was 33.5 MPa, and the porosity of the concrete was 14.3%. The hydrostatic pressure test procedure and sampling can be found in [41]. In order to maintain a stable solution concentration, the solution was changed every 24 h during the test. The chloride ion test of concrete was carried out following the Technical Code for Test and Inspection of Concrete for Water Transport Engineering (JTS/T 236-2019 [42]).

The hydrostatic pressure test device could perform the hydrostatic pressure test at a maximum of 2.5 MPa. Considering the hydrostatic pressure of coastal engineering and cross-sea engineering, and in order to compare test results, the hydrostatic pressures used in the test are 0, 0.3, 0.5, and 0.7 MPa, respectively.

Some samples, after a 24 h hydrostatic pressure test, were subjected to X-ray diffraction examination via a D8 ADVANCE A25 Powder X-ray Diffractometer (Bruker Physik-AG, Karlsruhe, Germany). The X-ray diffraction test adopted Cu-Kα; the voltage was 40 kV, the current was 30 mA, the scanning ranged from 10° to 45°, the scan speed was 10°/min, and the step was 0.02°. The samples used for X-ray diffraction were concrete powder after eliminating sand, and the samples were taken through a 0.15 mm sieve within a range of 4~8 mm from the intrusion surface.

## 4. Results

### 4.1. Effect of Hydrostatic Pressure on Chloride Ion Transport Behavior

**Figure 2:**
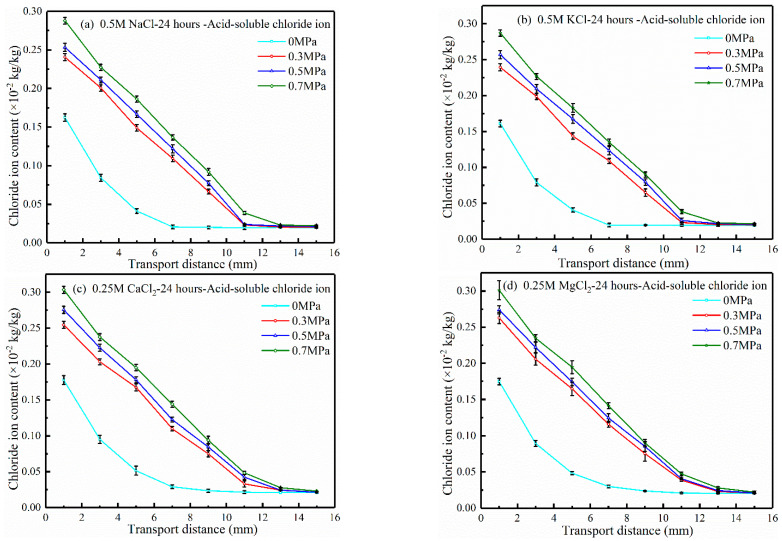
Chloride ion content profiles of concrete under different hydrostatic pressures.

Figure 2 shows the distribution of acid-soluble chloride ions in concrete under different hydrostatic pressures, which were applied for 24 h. Figure 2a–d present the test results of 0.5 M NaCl, 0.5 M KCl, 0.25 M CaCl_2_, and 0.25 M MgCl_2_ source solutions, respectively. It can be seen in Figure 2a–d that the chloride ion contents increase significantly in the presence of hydrostatic pressure. In the range of 0~2 mm, the chloride ion contents under hydrostatic pressures of 0.3, 0.5, and 0.7 MPa exceed those at zero pressure by about 40, 50, and 60%, respectively. It can be seen that the hydrostatic pressure accelerates the transport of chloride ions, and the higher the hydrostatic pressure, the faster the transport of chloride ions. The reason for the effect is related to the expansion and increment in size of the pores in concrete under hydrostatic pressure. Shang et al. [43] believed that the greater the hydrostatic pressure acting on the concrete surface, the larger the pore diameter within the influence range of the hydrostatic pressure, the easier the chloride ion passed through, and the greater the influence range of the hydrostatic pressure.

It can also be seen in Figure 2a–d that in four different source solutions, the chloride ion content profiles under hydrostatic pressures of 0.3, 0.5, and 0.7 MPa were approximately linear within a range of 0~11 mm from the intrusion surface. Under zero hydrostatic pressure, the transport distance of chloride ions was about 6 mm, 55% of that under 0.3 MPa hydrostatic pressure. The results show that the increase in hydrostatic pressure significantly accelerates chloride ion transport. Compared with the test results of 0.3 MPa, the transport distance of the chloride ion is increased by about 1~3 mm when the hydrostatic pressure is increased by 0.2 MPa. Thus, higher hydrostatic pressure corresponds to larger chloride ion transport distances. However, at any hydrostatic pressure value, the chloride ion contents are the same after a certain depth range, indicating that the effect of hydrostatic pressure has a limited application range. The reason is related to the distribution of pore water pressure in concrete under hydrostatic pressure. The pore water pressure decreases with the increase in the distance of the hydrostatic pressure surface, and the flow velocity decreases with the decrease in pore water pressure, so the transport velocity of the chloride ion also decreases [44].

To better characterize the effect of hydrostatic pressure on the chloride ion transport behavior, the original custom fitting function is used to fit the results in Figure 2 via Equation (2). The convective velocity v is determined by measuring the water penetration depth after the hydrostatic pressure test, and the value of this experiment is 2 × 10^−8^ m/s. The fitting results of the chloride ion apparent transport coefficient are listed in Table 2. It can be found in Table 2 that the chloride ion apparent transport coefficient of concrete under 0.3 MPa hydrostatic pressure exceeds that under 0 MPa hydrostatic pressure by 2.8~3.5 times. The chloride ion apparent transport coefficient increases about 2~8% for each 0.2 MPa increase in hydrostatic pressure, starting from 0.3 MPa. This is consistent with the results of the influence of hydrostatic pressure on chloride ion content distribution, and Jin et al. [21] believe that hydrostatic pressure accelerates the transport of the solution in concrete, and various ions are transported together with the solution.

### 4.2. Effect of Hydrostatic Pressure Action Time on Chloride Ion Transport Behavior

**Figure 3:**
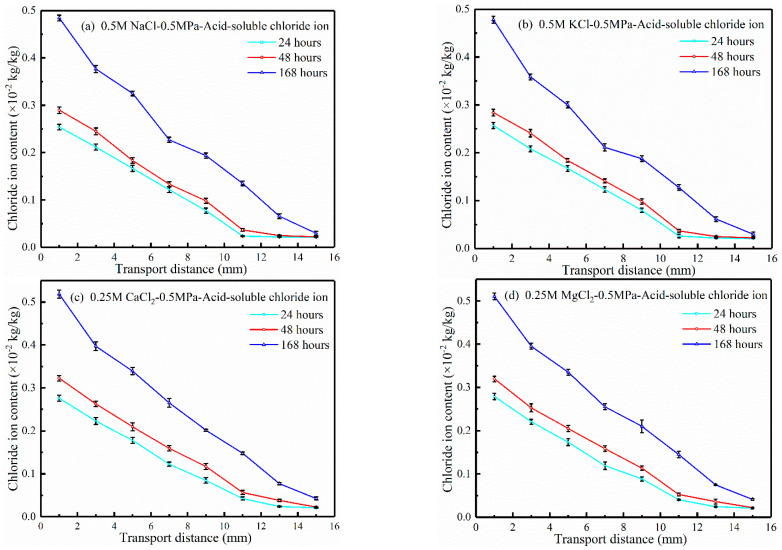
Chloride ion content profiles under 0.5 MPa hydrostatic pressure for different action times.

Once a marine underwater engineering structure is completed, hydrostatic pressure will continue. In addition to hydrostatic pressure magnitude, its action time also affects the chloride ion transport behavior. To investigate the effect of hydrostatic pressure action time on the chloride ion transport behavior, experiments were carried out on the concrete under 0.5 MPa hydrostatic pressure for 24, 48, and 168 h, respectively. The source solutions were 0.5 M NaCl, 0.5 M KCl, 0.25 M CaCl_2_, and 0.25 M MgCl_2_ source solutions, respectively. The chloride ion content profiles are shown in Figure 3.

As seen in Figure 3, the number of chloride ions entering concrete and their penetration depth increase with hydrostatic pressure action time. In monovalent cation solutions, chloride ions can be transferred 11 mm away from the invasion surface under hydrostatic pressure for 24 h, 13 mm for 48 h, and 15 mm for 168 h. In divalent cation solutions, 24 h is 13 mm, 48 h is 15 mm, and 168 h is more than 15 mm (limited by the sampling location and did not reach the hydrostatic pressure range). The results show that the transport distance of chloride ions increases with the increase in the hydrostatic pressure action time.

By comparing the chloride ion transport test of concrete in four cation solutions in Figure 3, it can be seen that under the same hydrostatic pressure action time, the chloride ion transport velocity in the solution where the two bivalent cations are located is faster than that in the solution where the two monovalent cations are located, and the chloride ion content in the concrete in the bivalent cation solution at the same transport distance is also higher. The reason is related to the cation radius. Ca^2+^ has a radius of 0.1 nm and the hydrated ion has a radius of 0.412 nm, Mg^2+^ has a radius of 0.066 nm and the hydrated ion has a radius of 0.428 nm, Na^+^ has a radius of 0.102 nm and the hydrated radius of 0.358 nm, and K^+^ has a radius of 0.133 nm and the hydrated ion has a radius of 0.331 nm [45]. In the same pore characteristics of the channel transport, the larger the ion radius, the greater the resistance of the channel, and the transport speed will be slower [12]. Chloride ion transport in the pore is also affected by the size of the hydrated ion radius. The hydrated ion radiuses of the two kinds of monovalent cations are basically equal, and the hydrated radius of the two kinds of divalent cations are basically equal, so it shows that the chloride ion apparent transport coefficient in the solution of the two kinds of cations with the same valence is not much different.

Equation (2) is used to fit the results in Figure 3, and the chloride ion apparent transport coefficients are calculated and listed in Table 3. It can be seen in Table 3 that the chloride ion apparent transport coefficients in the four kinds of solutions decrease with the increase in the hydrostatic pressure action time. The reason can be attributed to the retarding effect of the pore wall on the hydrostatic pressure. Under the action of the hydrostatic pressure, the solution entering concrete pores will be reduced by the effect of pore resistance. The influence range of hydrostatic pressure on chloride ion transport is related to the hydrostatic pressure. Although the hydrostatic pressure action time increases, the solution transport enters a stable diffusion state under the hydrostatic pressure, so the chloride ion apparent transport coefficient decreases with the increase in time. According to the studies by Mejibro [46] and Maage [47,48], the decrease in the chloride ion apparent transport coefficient may also be related to the influence of cement hydration and chloride ion binding and adsorption on the pore thinning and blocking of concrete.

**Figure 4:**
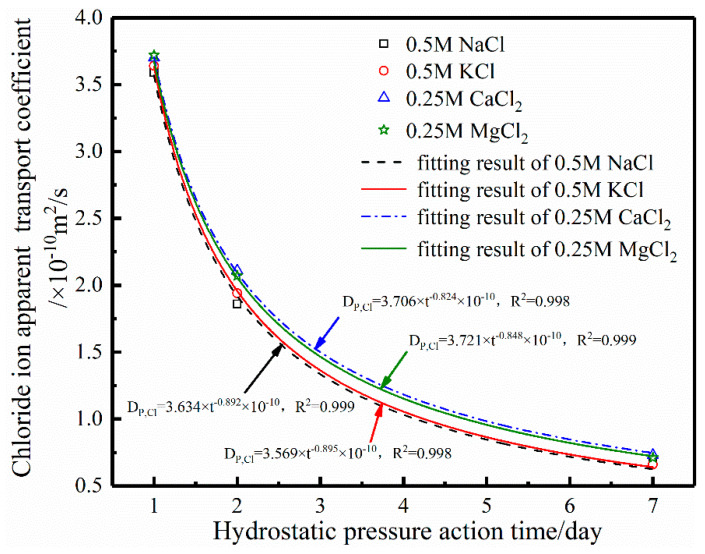
Fitting results of the chloride ion apparent transport coefficient and hydrostatic pressure action time.

Based on the results in Table 3, the relationship between the chloride ion apparent transport coefficient and hydrostatic pressure action time is fitted, as shown in Figure 4. As can be seen from the fitting results in Figure 4, the correlation coefficient between the chloride ion apparent transport coefficient in the four solutions and the fitting results between the hydrostatic pressure action time of 0.5 MPa exceeds 0.99, indicating that the fitting results are good. It can also be seen in Figure 4 that the fitting curves corresponding to the two monovalent cations are basically overlapping, and the fitting curves corresponding to the two divalent cations are also basically overlapping. It can be seen that the chloride ion transport results in the cation solution with the same valence are basically consistent. Liu et al. [49] and Pan et al. [50] explained and analyzed the consistent behavior of the influence of monovalent or bivalent cations on the chloride ion transport behavior from the radius of the cation, pH of the concrete pore liquid, electronegativity of the chloride ion, and the theory of neutral balance.

### 4.3. Effect of Cation Type on Chloride Ion Transport Behavior

**Figure 5:**
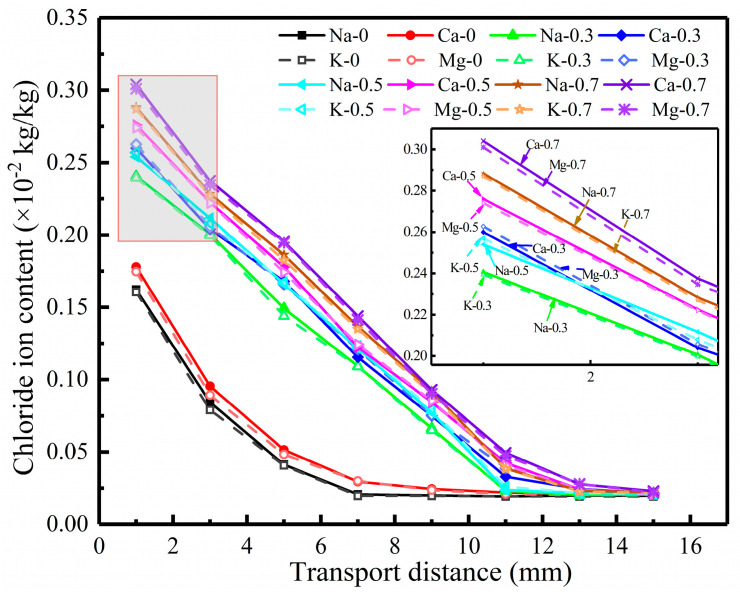
Chloride ion content profiles of concrete under different hydrostatic pressures in NaCl and CaCl_2_ source solutions (the cation in the code represents the type of solution, and the number behind the short horizontal line represents the hydrostatic pressure).

Figure 5 shows the chloride ion content profiles of concrete under different hydrostatic pressures in the four source solutions. As shown in Figure 5, under the same experimental conditions, the distribution curve of chloride ion content in the source solution corresponding to the same valence cations basically overlaps, indicating that the source solution with the same valence of cations has little influence on the transport behavior of chloride ions. The concrete chloride ion content in the source solution corresponding to the divalent cation is higher than that in the solution corresponding to the monovalent cation, and the test results show that the cation valence affects chloride ion transport behavior. It can be seen in Table 2 that under the conditions of 0.3, 0.5, and 0.7 MPa, the relative deviation between the chloride ion apparent transport coefficients of concrete in the two kinds of monovalent cation solutions under the same hydrostatic pressure is up to 1.4%, and that in the two kinds of divalent cation solutions is 1%. In Table 3, under the same hydrostatic pressure action time, the relative deviation between the chloride ion apparent transport coefficients in the two kinds of monovalent cation solutions is 4.2%, and that in the two kinds of divalent cation solutions is 1.9%. It can be seen that under the same conditions, the maximum deviation of the influence results on chloride ion transport is less than 5% when the valence of the cations is the same. The results show that when the valence of the cations in the solution is the same, the effects of the cations with the same valence on chloride ion transport are similar. Liu et al. [38] reached the same conclusion when studying the chloride ions transport behavior in four different chloride salt solutions in fly ash concrete. The test results show that in multi-source cation solutions, the cations with the same valence are regarded as the same amount as the single cation solutions with the same valence so as to simplify the analysis of the affecting factors of the chloride ion transport behavior.

### 4.4. Calculation Model of the Chloride Ion Apparent Transport Coefficient

**Figure 6:**
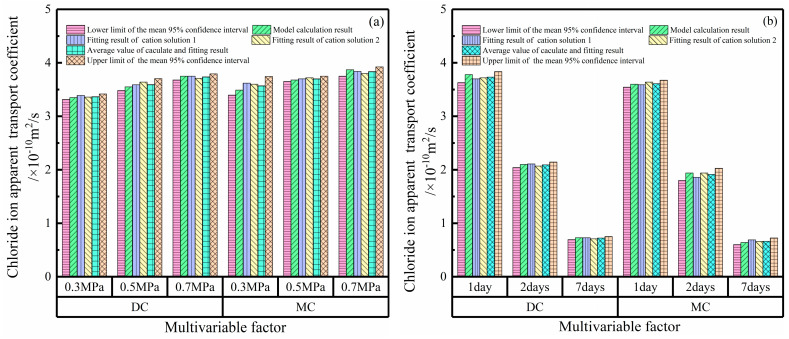
Deviation analysis results of the chloride ion apparent transport coefficient: (**a**) analysis results under different hydrostatic pressures; (**b**) analysis results of different hydrostatic pressure action times (DC, divalent cation; MC, monovalent cation).

According to the analysis in Section 4.3, cations with the same valence have little influence on chloride ion transport behavior, while cations with different valence have different influence on chloride ion transport behavior, which shows that the chloride ion transport coefficient in the solution of the divalent cation solution is larger than that in the solution of the monovalent cation solution. According to the analysis results in Section 4.3, cations with the same valence can be replaced by cations with the same single valence. In order to quantitatively analyze the influence of cation type on chloride ion transport behavior, based on chloride ion apparent transport coefficients and hydrostatic pressures in Table 2 and hydrostatic pressure action times in Table 3, taking 0.5 M NaCl as the reference solution, and considering the cation type influence coefficient, the calculation models of the chloride ion apparent transport coefficient were expressed via Equations (3) and (4), respectively.

(3) $$D_{P,Cl}=(\frac{P}{k_{ca}}+3.05\cdot k_{ca})\times 10^{-10}(m^{2}/s)$$

(4) $$D_{P,Cl}=k_{ca}\cdot a_{s}\cdot t^{-\lambda /k_{ca}}$$

where *D_P,Cl_* is the chloride ion apparent transport coefficient, m^2^/s; P is the value of the applied hydrostatic pressure, MPa; k_ca_ is the cation type influence coefficient, and since the 0.5 M NaCl solution is the reference solution, *k_ca_* = 1, while in the divalent cation solution, combined with the fitting results of the chloride ion apparent transport coefficients in Table 2 and Table 3, *k_ca_* in this study is 1.05; *t* is hydrostatic pressure action time, day; *a_s_* is the regression coefficient of the reference solution (the reference solution of this study is a 0.5 M NaCl solution), dimensionless; and λ is the regression coefficient and is dimensionless. Substituting 0.3, 0.5, and 0.7 MPa into Equation (3), the chloride ion apparent transport coefficients in the monovalent cation solution were obtained as 3.35 × 10^−10^, 3.55 × 10^−10^, and 3.75 × 10^−10^ m^2^/s, and the chloride ion apparent transport coefficients in the divalent cation solution were obtained as 3.49 × 10^−10^, 3.68 × 10^−10^, and 3.87 × 10^−10^ m^2^/s. Figure 6a shows the calculated results in Equation (3) and the fitting results in Table 2, as well as the mean values of the calculated and fitted results and their 95% confidence intervals. It can be seen from the results in Figure 6a that the calculated results of the model are within the 95% confidence interval of the average value, which indicates that the calculated results of the calculation in Equation (3) between the hydrostatic pressure and chloride ion transport coefficient modified by the cation type influence coefficient with a 0.5 M NaCl solution as the reference solution are credible. This can be used to predict the chloride ion transport coefficient of marine concrete with the same concrete mix and solution type under different hydrostatic pressures.

Similarly, based on the fitting results in Figure 4, relevant data were substituted into Equation (4), and the relationship between the hydrostatic pressure action time and the chloride ion apparent transport coefficient was adjusted using the cation type influence coefficient, which can be expressed by Equation (5).

(5) $$D_{P,Cl}=3.6\cdot k_{ca}\times t^{-0.89/k_{ca}}\times 10^{-10}(m^{2}/s)$$

The chloride ion apparent transport coefficients in the divalent cation solution calculated via Equation (5) are 3.78 × 10^−10^, 2.10 × 10^−10^, and 0.73 × 10^−10^ m^2^/s for 1, 2, and 7 d, respectively. The chloride ion apparent transport coefficients in the monovalent cation solution calculated via Equation (5) are 3.6 × 10^−10^, 1.94 × 10^−10^, and 0.64 × 10^−10^ m^2^/s for 1, 2, and 7 d, respectively. Figure 6b shows the calculated results in Equation (5) and fitting results in Table 3, as well as the mean values of the calculated and fitted results and their 95% confidence intervals. It can be seen from the results in Figure 6b that the calculated results of the model are within the 95% confidence interval of the average value, which indicates that the calculated results of the calculation in Equation (5) between the hydrostatic pressure and the chloride ion transport coefficient modified by the cation type influence coefficient with a 0.5 M NaCl source solution as the reference solution are credible. This can be used to predict the chloride ion transport coefficient of marine concrete under the same conditions in different periods.

### 4.5. Effect of Hydrostatic Pressure on Chloride Ion Binding Capacity

When chloride ions moved through concrete pores, they were easily absorbed and bound by the hydration products of concrete. The ability of hydration products to adsorb and bind chloride ions is commonly characterized by the chloride ion binding capacity β.

(6) $$\beta =\frac{C_{as}-C_{ws}}{C_{as}}\times 100\%$$

where *C_as_* and *C_ws_* refer to concentrations of acid-soluble and water-soluble chloride ions, *g*/*g*.

**Figure 7:**
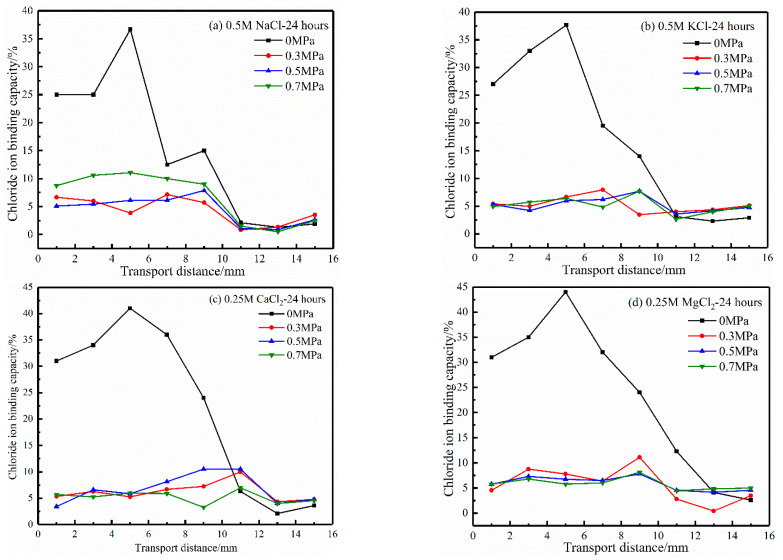
Effect of hydrostatic pressure on the chloride ion binding capacity.

The more chloride ions are absorbed and bound by hydration products, the fewer free chloride ions, and the better the durability of concrete. Figure 7 shows the relationship between the chloride ion binding capacity and chloride ion transport distance of concrete under different hydrostatic pressures in four cation source solutions. As can be seen from the results of the chloride ion binding capacity of the four cation source solutions in Figure 7, under hydrostatic pressures of 0.3, 0.5, and 0.7 MPa, the binding capacity of the chloride ions is about 0~15%, while under 0 MPa, it was about 0~40%. In the four cation source solutions, the binding capacity of chloride ions shows the same change rule under the action of hydrostatic pressure, and the greater the hydrostatic pressure, the smaller the binding capacity of chloride ions. The results show that the existence of hydrostatic pressure reduces the binding capacity of chloride ions. Song et al. [34] showed that the chloride ion binding capacity was about 40~70% in the natural diffusion experiment. The chloride ion binding capacity under hydrostatic pressure was about 10~20% of that of the natural diffusion experiment. An earlier study by Jin et al. [21] also revealed that the chloride ion binding capacity under 0.2 MPa hydrostatic pressure was between 0 and 12%, which was close to the 10~20% range of the natural diffusion experiment.

**Figure 8:**
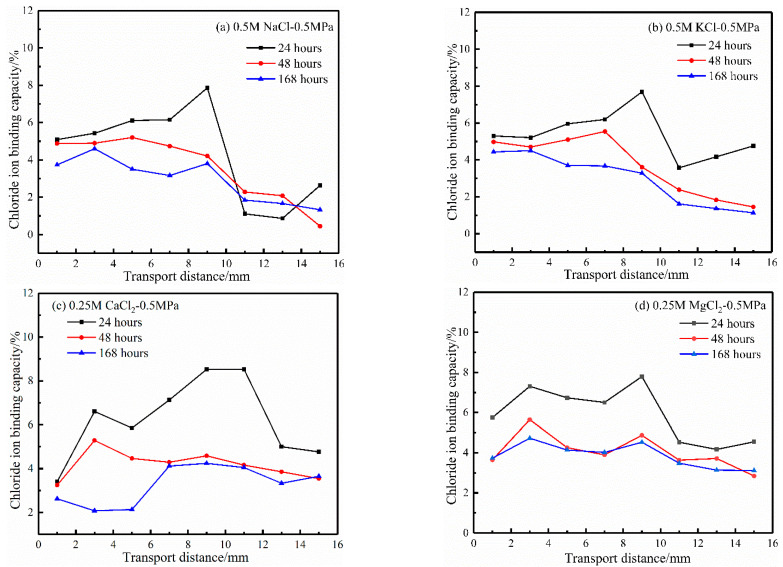
Effect of hydrostatic pressure action time on the chloride ion binding capacity.

Figure 8 displays the relationship between the chloride ion binding capacity and transport distance in four cation source solutions under different hydrostatic pressure action times. It can be seen from the results of the chloride ion binding capacity in the four cation source solutions in Figure 8 that the chloride ion binding capacity decreases with hydrostatic pressure action time. The longer the action time of hydrostatic pressure, the lower the chloride binding capacity. The results may be because the number of binding chloride ions increases with hydrostatic pressure action time. Still, the amount of total chloride ions increases faster, which reduces the binding capacity of chloride ions [10].

### 4.6. XRD

**Figure 9:**
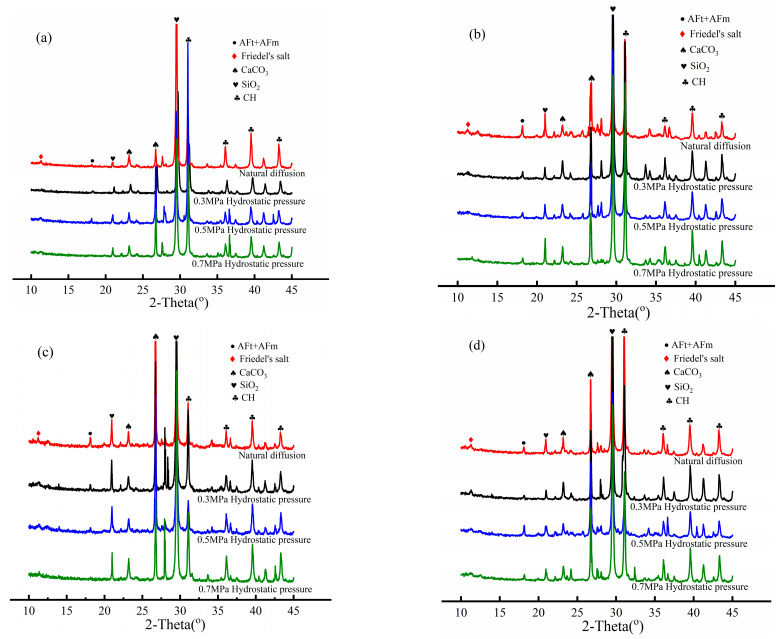
XRD patterns of concrete samples: (**a**) NaCl source solution; (**b**) KCl source solution; (**c**) CaCl_2_ source solution; (**d**) MgCl_2_ source solution.

Figure 9 shows the XRD patterns of concrete in four cation source solutions. It can be observed that the intensity of Friedel’s salt diffraction peak under hydrostatic pressure is lower than that of the natural diffusion experiment, and the intensity of Friedel’s salt diffraction peak decreases with the increase in hydrostatic pressure in the same solution. Thus, the chloride ion binding capacity of concrete under hydrostatic pressure differs from that under natural diffusion, and high hydrostatic pressure reduces the chloride ion binding capacity. When hydrostatic pressure is applied, the solution containing chloride ions is transported into concrete under a hydrostatic pressure gradient [10,13]. Hydrostatic pressure accelerates the flow velocity of the solution in the pores of concrete, reducing the number of chloride ions absorbed by the pore walls. Hence, the chloride ion binding capacity decreases [41].

Comparing the XRD results of concrete in the solution of four sources in Figure 9, Figure 9a,b show the XRD of concrete in two monovalent cation solutions, and it is found that there is no significant difference in the peak value of F salt diffraction. Similarly, the XRD results of the concrete in the divalent cation solutions in Figure 9c,d show little difference in the diffraction peak of F salt. By comparing Figure 9a,b with Figure 9c,d, it is found that the peak value of F salt in the XRD results of the concrete in two divalent cation solutions is slightly higher than that in the XRD results of the concrete in two monovalent cation solutions. The test results show that under the same conditions, the adsorption capacity of bivalent cations is higher than that of monovalent cations, which is consistent with the results of the natural diffusion test in the literature [32,34]. It can be seen from the XRD peak value in Figure 9 that the peak value is small. The reason for this result is related to the selected location of samples for the XRD test and the short time for chloride ion transport. The XRD samples selected in this experiment are powders that are 4–8 mm away from the concrete surface. During the hydrostatic pressure test period for 24 h, the chloride ion content in the samples is less and the amount of F salt generated is less, resulting in a smaller peak value of F salt in the test results.

## 5. Discussion

**Figure 10:**
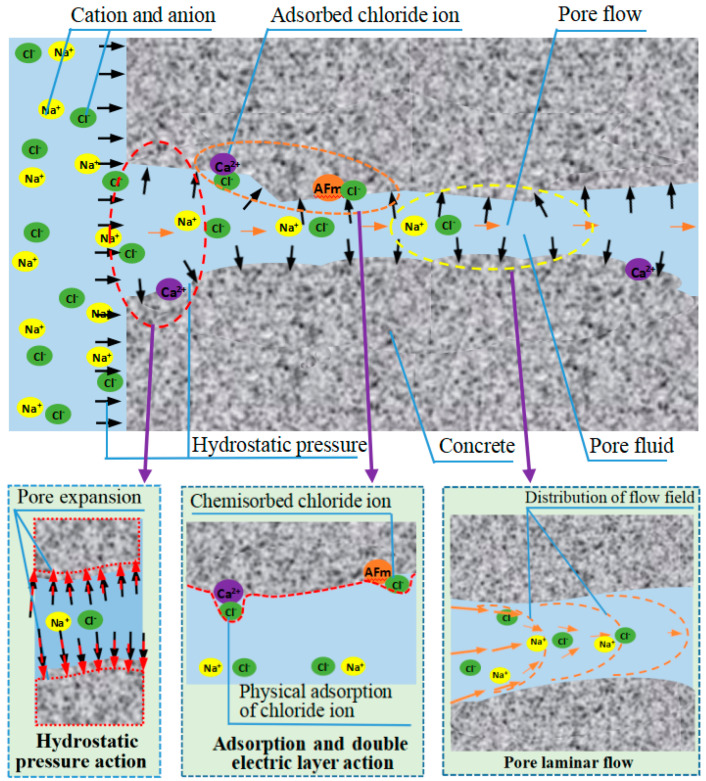
Schematic of the influence of hydrostatic pressure on the chloride ion transport in concrete.

The above results prove that hydrostatic pressure accelerates the transport rate of chloride ions in concrete, changes the adsorption and binding characteristics of hydration products to chloride ions, and increases the number of free chloride ions in marine concrete. The effect mechanism of hydrostatic pressure on the chloride ion transport process of concrete is shown in Figure 10. The influence of hydrostatic pressure on chloride ion transport is related to the change in the microstructure of concrete and the pore liquid transport process by hydrostatic pressure. The expansion effect of hydrostatic pressure makes the original pore diameter of concrete increase, and excessive hydrostatic pressure even produces micro-cracks in concrete. The larger the pore diameter, the more micro-cracks, the smaller the transport resistance of chloride ions, and the transport rate and transport quantity of chloride ions increase. In addition, the hydrostatic pressure increases the transport rate of pore liquid, and the pore liquid carries chloride ions in the pores together. The greater the hydrostatic pressure, the more chloride ions are transferred into the concrete along with the pore liquid, and the faster the transport rate. The hydrostatic pressure not only increases the pore diameter but also increases the chloride ion transport rate. According to the Hagen–Poeuille law [51], in the porous seepage field, the flow velocity in the pore center is large and the surrounding area is low, and the hydrostatic pressure increases the chloride ion quantity in the pore center, thus affecting the adsorption of chloride ions by the pore wall and reducing the adsorption and binding amount of chloride ions by hydration products.

In addition, the hydrostatic pressure decreases along the path due to the friction resistance of the inner wall of the pore, the change in pore diameter, and the change in direction; that is, the hydrostatic pressure decreases with the increase in the transport path until it decreases to zero or penetrates the transmission path. Therefore, under certain pore characteristics, the influence range of the hydrostatic pressure on chloride ion transport behavior increases with the hydrostatic pressure. Because the hydrostatic pressure decreases along the pore, the influence of the hydrostatic pressure on the chloride ion transport becomes smaller and smaller along the distance from the surface of the hydrostatic pressure.

Finally, different types of cations have different ionic radii. Ca^2+^ has a radius of 0.1 nm and the hydrated ion has a radius of 0.412 nm, Mg^2+^ has a radius of 0.066 nm and the hydrated ion has a radius of 0.428 nm, Na^+^ has a radius of 0.102 nm and the hydrated radius of 0.358 nm, and K^+^ has a radius of 0.133 nm and the hydrated ion has a radius of 0.331 nm [45]. When the pore is constant, the smaller the radius of the cation, the smaller the influence of the pore size on the ion transport in the pore, and the faster the rate. When chloride ions are transported in pores, in order to maintain the local electric neutral balance, cations with equal charges are needed to travel together. Different types of cations in the source solution have different accompanying cation radii. The smaller the accompanying cation radius, the less influence it has on the chloride ion transport process, and the faster the chloride ion transport rate [37,49]. The radius of divalent cations is less than the radius of monovalent cations, so the chloride ions in the divalent cation source solutions transport faster than those in the monovalent cation source solutions under the same conditions. In addition, the pore interface of concrete is generally negatively charged; in order to maintain a neutral balance, the pore wall often adsorbs or binds the cation in the pore liquid, and the cation will adsorb the anion, which not only forms a double electric layer effect but also affects the transport of chloride ions. Due to the different types of cations in the source solution, the zeta potentials in the double electric layer are also different, and the ability to adsorb chloride ions is different. The electronegativity of divalent cations is greater than that of monovalent cations, the bivalent cations have strong adsorption capacities for chloride ions, and the influence on the transport of chloride ions is greater than that of monovalent cations. After absorbing ions, the boundary size of the original pore wall is changed, and a pore barrier is formed in the pore, which hinders the passage of subsequent ions.

## 6. Conclusions

This study investigated the effects of hydrostatic pressure and hydrostatic pressure action time on chloride ion transport behavior in four cation source solutions using a self-developed test device. The following conclusions are drawn.

In marine underwater engineering, hydrostatic pressure is the main driving force for chloride ions to transport into concrete. The larger the hydrostatic pressure, the more chloride ions will be transported into concrete, and the deeper they will be transported. The chloride ion apparent transport coefficient increases linearly with the increase in hydrostatic pressure, on the basis of 0.3 MPa, the chloride ion apparent transport coefficient increases by 2–8% with the increase of 0.2 MPa hydrostatic pressure. An empirical calculation model of hydrostatic pressure and the chloride ion apparent transport coefficient is established.

The longer the hydrostatic pressure is applied, the more chloride ions penetrate the concrete, and the deeper the transport distance. The chloride ion apparent transport coefficient decreases exponentially with the hydrostatic pressure action time. An empirical model for calculating the hydrostatic pressure action time and the chloride ion apparent transport coefficient is established.

The cation type in the source solution influences the chloride ion transport behavior, and the chloride ion transport rate is about 5% higher in divalent cation source solutions than in monovalent cation source solutions. The empirical models of hydrostatic pressure, hydrostatic pressure action time, and the chloride apparent transport coefficient are modified by introducing the influence coefficient of cation type.

Hydrostatic pressure reduces the chloride ion binding capacity of concrete, and the chloride ion binding capacity under hydrostatic pressure is 10~20% of that in the natural diffusion test.

## Figures and Tables

**Table 1:** Chemical compositions of cement (wt.%).

Compositions	CaO	SiO_2_	Al_2_O_3_	Fe_2_O_3_	MgO	Na_2_O	K_2_O	SO_3_	LOI
Contents	59.8	26.4	3.87	3.45	1.61	0.30	0.22	2.56	2.80

**Table 2:** Chloride ion apparent transport coefficients under different hydrostatic pressures.

Solution Types	Chloride Ion Apparent Transport Coefficient (×10^−10^ m^2^/s)			
	0 MPa	0.3 MPa	0.5 MPa	0.7 MPa
NaCl	1.03 (0.93)	3.39 (0.99)	3.59 (0.98)	3.75 (0.99)
KCl	0.97 (0.92)	3.36 (0.99)	3.64 (0.98)	3.71 (0.99)
CaCl_2_	1.30 (0.92)	3.62 (0.99)	3.70 (0.99)	3.84 (0.99)
MgCl_2_	1.23 (0.93)	3.60 (0.99)	3.72 (0.99)	3.80 (0.99)

Note: The figures in brackets in the table are the results of fitting the correlation coefficient (R^2^).

**Table 3:** Chloride ion apparent transport coefficients under different hydrostatic pressure action times.

Solution Types	Chloride Ion Apparent Transport Coefficient (×10^−10^ m^2^/s)		
	24 h	48 h	168 h
0.5M NaCl	3.59 (0.98)	1.86 (0.99)	0.69 (0.99)
0.5M KCl	3.64 (0.98)	1.94 (0.98)	0.66 (0.99)
0.25M CaCl_2_	3.70 (0.99)	2.11 (0.99)	0.73 (0.99)
0.25M MgCl_2_	3.72 (0.99)	2.07 (0.99)	0.71 (0.99)

Note: The figures in brackets in the table are the results of fitting the correlation coefficient (R^2^).

## Data Availability

The raw data supporting the conclusions of this article will be made available by the authors on request.
